# A case report of refractory amebic colitis and literature review

**DOI:** 10.1097/MD.0000000000037195

**Published:** 2024-02-09

**Authors:** Yupei Shao, Hong Lv, Weixun Zhou, Baotong Zhou, Qingwei Jiang, Jiaming Qian

**Affiliations:** a Department of Gastroenterology; b Department of Pathology; c Department of Infectious Diseases, Peking Union Medical College Hospital, Chinese Academy of Medical Science & Peking Union Medical College, Beijing 100730, China.

**Keywords:** amebic colitis, diloxanide, inflammatory bowel disease, metronidazole

## Abstract

**Rationale:**

Amebic colitis has been less prevalent in recent times in China, and the similarity of its symptoms to those of inflammatory bowel disease (IBD) results in the difficulty of early identification and diagnosis.

**Patient concerns:**

A 31-year-old male who exhibited intermittent diarrhea and hematochezia was highly suspected as IBD initially. Despite the partial relief of symptoms following the administration of mesalamine, the endoscopic ulcers remained largely unchanged.

**Diagnoses:**

Two years after the onset of mesalamine therapy, amebic cysts were detected in stool microscopy and trophozoites were found on the surface of cecal ulcers. The patient was then diagnosed with amebic colitis.

**Interventions:**

After 2 rounds of standardized metronidazole treatment, amebic colitis remained refractory until diloxanide was administered.

**Outcomes:**

The patient remained asymptomatic, and the mucosa of colon was normal during the annual follow-up.

**Lessons:**

Individuals newly diagnosed with IBD should undergo essential screening for amebiasis. And the use of steroids should be taken with caution, especially in cases where the effect of mesalamine is limited. For symptomatic intestinal amebiasis, even after the administration of tissue amebicides, the continued use of luminal amebicides is necessary to prevent recurrence.

## 1. Introduction

Intestinal amebiasis is an intestinal disease caused by the invasive parasite *Entamoeba histolytica*. Although 90% of amebiasis cases are asymptomatic, *E. histolytica* is a predominant cause of severe diarrhea worldwide.^[1]^ There are 2 stages of *E. histolytica*: the infectious cystic form and the invasive trophozoite form.^[2]^ People get infected mostly via oral–fecal route, through ingestion of food or water contaminated by amebic cysts. Moreover, oral–genital or oral–anal contact among homosexuals are also important ways of transmission in developed countries. Amebic cysts pass through the gastrointestinal tract and transform into trophozoites in the terminal ileum or proximal colon by adhering to colonic mucins. Through disrupting the mucoepithelial barrier, trophozoites subsequently invade the intestinal mucosal layer to form flask-shaped ulcers.^[3]^

The overall worldwide prevalence of Entamoeba infection in humans is 3.6%,^[4]^ but amebiasis is mostly endemic in developing countries with poor hygiene and nutrition conditions, such as India, Africa, Mexico, and some parts of South America.^[5]^ In developed counties, although the incidence of amebiasis is relatively low, inflammatory bowel disease (IBD) is prevalent. Amebic colitis is defined as symptomatic intestinal amebiasis presenting with diarrhea, abdominal pain and weight loss, and multiple ulcers in endoscopy.^[6]^ Due to the resemblance of clinical features between amebic colitis and IBD, there is a high risk of misdiagnosis.^[6,7]^ Therefore, a cautious approach is necessary when evaluating patients exhibiting symptoms of IBD, especially in regions where amebiasis is prevalent. Here, we report a case of refractory amebic colitis, which was initially suspected as IBD. After 2 rounds of standardized metronidazole (MTZ) treatment, intestinal ulcers were still present on colonoscopy and amebic cysts could be repeatedly found in stool examination until the administration of diloxanide. A comprehensive review of intestinal amebiasis is discussed with focus on its differentiation from IBD and the standardized treatment.

## 2. Case report

**Figure 1.**
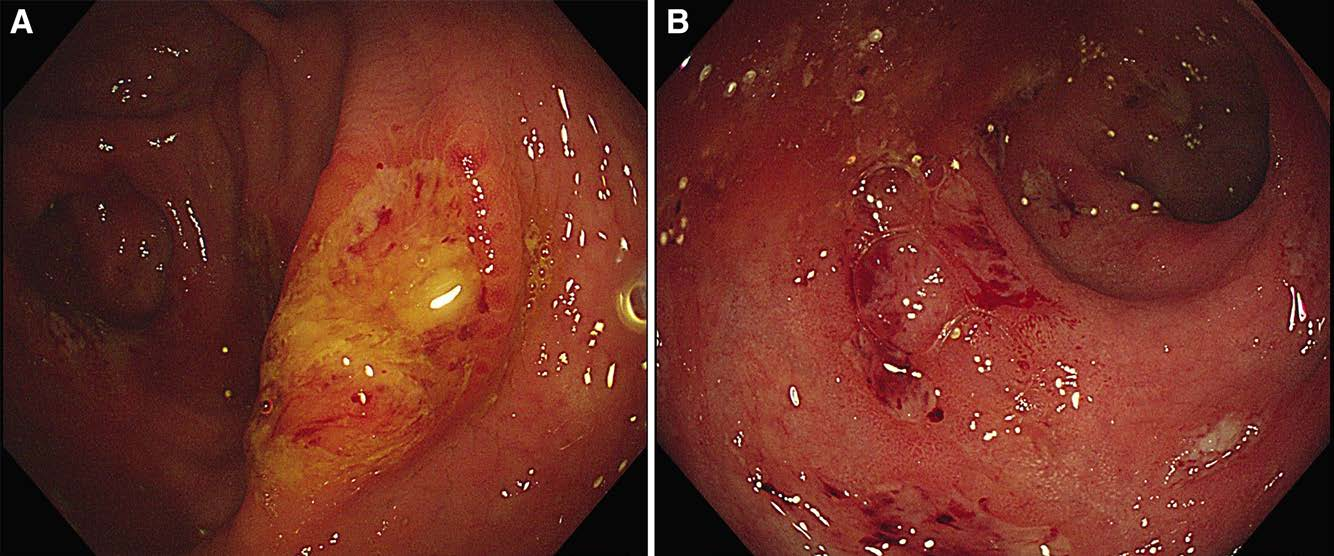
The multiple irregular ulcers covered with white or yellow exudates were observed in cecum (A) and rectum (B), and the rectum mucosa showed diffuse congestion and slightly blurred vascular texture.

A 31-year-old, previously healthy male was admitted to our hospital for a 5-month history of intermittent diarrhea and hematochezia occurring 3 to 5 times per day, without fever, abdominal pain, and weight loss. The patient was a bus driver residing in a northern city of China with no history of consuming raw meat. There was no travel history or specific medicine administration before disease onset. Routine physical examination and blood tests were basically normal. Fecal occult blood test was positive, and no enteropathogens were identified in stool analysis. Colonoscopy demonstrated multiple irregular ulcers with diameters of 2 to 5 mm in the cecum, opening of appendix and rectum (Fig. 1). The superficial ulcers were covered with white or yellow exudates and mucosa between ulcers were relatively normal. The rectum mucosa showed diffuse congestion and slightly blurred vascular texture. Endoscopic biopsies showed mild chronic colitis with inflammatory exudate. On computed tomography scan, localized thickening of the intestinal wall could be seen from terminal ileum to rectum.

A diagnosis of IBD was suspected, and the patient was initiated on regular mesalamine therapy. Abdominal symptoms were partially relieved, but intestinal ulcers remained essentially unchanged after 1 year of treatment. Moreover, architectural alterations of the crypt, mild cryptitis, and crypt abscess were newly observed in the rectal mucosa. Two years after the onset of mesalamine therapy, amebic trophozoites and cysts were found in stool microscopy during the follow-up. But no amebic trophozoites had been observed in the past 4 examinations of endoscopic biopsies. Treatment was switched to MTZ: 600 mg orally, 3 times per day for 14 days. Four weeks after the initiation of MTZ, abdominal symptoms and endoscopic ulcers were completely relieved.

**Figure 2.**
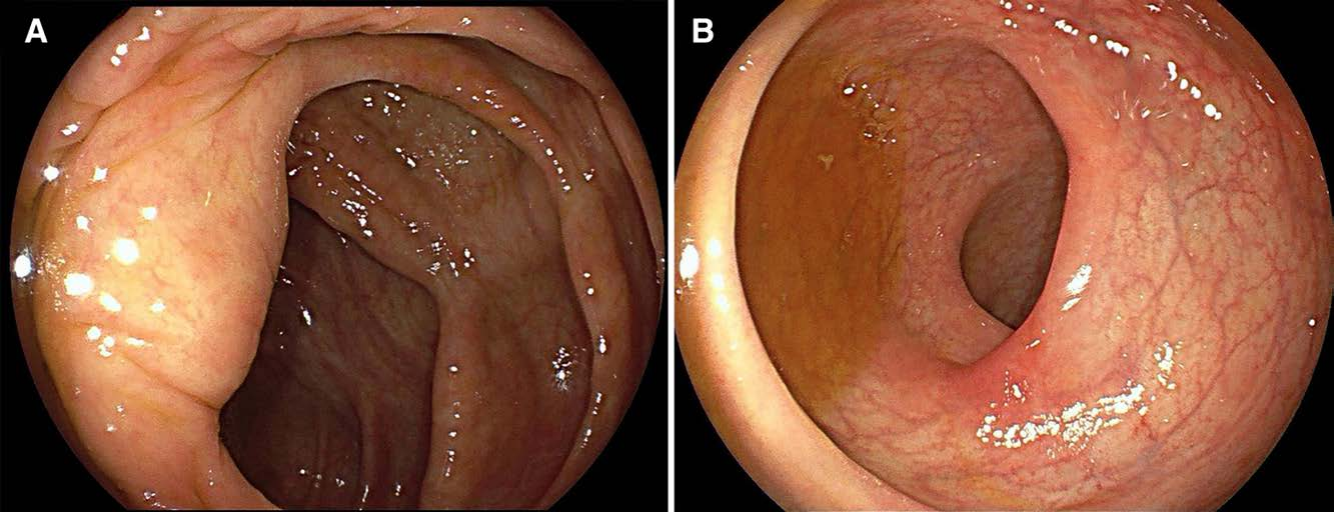
The mucosa of colon was completely normal, and no ulcers was observed in cecum (A) and rectum (B).

However, after 1 year of the initial antiamebiasis treatment, the intestinal ulcers recurred and amebic trophozoites were found on the surface of cecal ulcers. The patient underwent a second round of MTZ treatment. But the effect was limited, and amebic cysts could still be detected in the stool. Despite receiving standard antiamebiasis treatment, the intestinal disease remained refractory. We considered the possibility of concurrent IBD or amebic involvement in other organs. A multidisciplinary treatment was employed to analyze the clinical, histopathological, and radiological information of this patient. A new computed tomography scan was performed, and it showed slight thickening of the ileocecal and rectal walls, with no evidence of amebic involvement in other organs. Furthermore, the intestinal pathology exhibited solely chronic inflammation with mild crypt architectural distortion. Among 7 pathological examinations, only 1 crypt abscess was observed. Considering the complete resolution of abdominal symptoms after MTZ treatment, the multidisciplinary treatment considered no evidence of IBD. The administration of diloxanide (500 mg orally, 3 times per day for 14 days) was followed to eliminate the luminal cyst. The patient underwent the colonoscopy 1 year after the treatment of diloxanide, and no ulcers were identified and the mucosa of colon was normal (Fig. 2). During the annual follow-up, the stool analysis and endoscopic biopsies with periodic acid–Schiff (PAS) stain did not identify any entamebae, and fecal occult blood test was negative.

## 3. Discussion

Here we described a case of refractory amebic colitis, which was initially suspected as IBD and repeatedly recurrent due to the absence of sequential luminal amebicides following MTZ treatment. There is a paucity of data about the overall prevalence of *E. histolytica* in China, but Chinese Center for Disease Control and Prevention has reported that the incidence cases of amebic dysentery in China decreased from 3308 in 2014 to 775 in 2019.^[8]^ And southern provinces, especially southwestern provinces, are the most prevalent region of amebic dysentery. Due to the lower prevalence and its symptom similarity with IBD, early recognition of amebic colitis is becoming increasingly challenging.

Stool microscopy is considered as the gold standard in the diagnosis of amebic colitis, and it remains the most widely used test. However, this method is susceptible to variables of stool like storage condition, retention quality, and processing time,^[9]^ and the sensitivity of it is only 60%.^[10]^ Other nonpathogenic Entamoeba species including *E. dispar, E. moshkovskii, E. hartmanni*, and *E. bangladeshi* can hardly be distinguished from *E. histolytica* in morphology by microscopy.^[11]^ In this patient, the results of stool parasite were negative within the first 3 years of disease onset. Enzyme-linked immunosorbent assay tests have been developed to detect serologic anti-ameba antibodies or fecal antigens. However, serological anti-ameba antibody is difficult to distinguish past and present infections.^[12]^ And the result of antibody detection would be negative in cases of amebic colitis without tissue invasion. Fecal antigen testing is relatively simple and rapid, and it can differentiate *E. histolytica* from *E. dispar*, resulting in the development of a variety of commercial kits. Molecular detection based on the polymerase chain reaction has been increasingly important in the identification of Entamoeba species due to the high sensitivity and specificity.^[13]^ At present, real-time polymerase chain reaction is considered the most sensitive method to identify *E. histolytica*. Colonoscopy with histological examination is prevalent and essential in the diagnosis of amebic colitis. The typical endoscopic appearance of amebiasis is multiple punctuate ulcers with a diameter of 2 to 10 mm. The ulcers are relatively small and superficial and mostly distributed in the cecum and rectum.^[14]^ Nagata et al identified that the presence of cecal lesions, multiple lesions, and exudate were independent predictive endoscopic features of amebic colitis,^[15]^ which was consistent with our patient.

A retrospective study from China found that all 16 amebic colitis can be detected with trophozoites from their biopsy specimens, indicating that histological examination is essential for diagnosis.^[14]^ However, amebic trophozoites were mainly discovered on the surface of mucosa or in exudates. But pathologists normally paid too much attention on the mucosa structure and ignored the exudates. Besides, some specimens truly contained limited number of trophozoites due to the random distribution. Therefore, a negative biopsy result cannot exclude the diagnosis of amebic colitis, and all patients from that Chinese study conducted repeated endoscopy examination. In this patient, the positivity rate of trophozoites in mucosal biopsy was 16.67%, which was ultimately detected in the surface mucosa of the cecal ulcer. Immunohistochemistry or PAS stain can also assist in the identification of amebic cysts or trophozoite.^[16]^ In this study, PAS stain was used to evaluate the efficacy of treatment to ensure that there were no residual ameba cysts.

We misdiagnosed this patient as IBD at disease onset and failed to correctly identify the condition for 2 years. However, during our follow-up, we closely monitored stool enteropathogens and exercised caution in the use of steroids when there was no significant improvement in intestinal ulcers, thus avoiding complications such as intestinal perforation. At first diagnosis, 75% of amebic colitis patients were identified as IBD,^[14]^ which may result in the administration of steroids and fulminant colitis. In a systematic review of patients with amebic colitis, administration of steroids was a risk factor of fulminant colitis and can result in rapid progression of disease. Despite all fulminant amebic colitis patients received treatment with MTZ, nearly 50% of patients underwent surgical intervention and 25% of patients died.^[17]^ What makes the situation more complicated is that IBD population may combine with concurrent amebic colitis. Compared with normal people, patients with IBD were reported to have a higher prevalence of amebiasis colitis (16% vs 1.7%).^[18]^ The endoscopic and pathological characteristics of colonic ulcers can assist to distinguish amebic colitis and IBD. Compared to IBD, ulcers of amebic colitis are mostly superficial and generally covered with inflammatory exudates,^[14]^ and the degree of inflammation and structure disorder is less severe. In a Chinese study, 80% amebic colitis patients were found with crypt architectural alteration, but mostly were mild; 75% had cryptitis locating in the superficial layer of mucosa; and none of them had crypt abscess.^[14]^ In this case, crypt architectural distortion and cryptitis were found in almost 80% of endoscopic biopsies, and only one crypt abscess was identified.

All patients with *E. histolytica* infection, even those asymptomatic, should receive treatment to prevent progression. Carrero et al summarized the current therapeutic options of amebic colitis.^[1]^ Among all the drugs available, MTZ remains the first choice and is widely prescribed for the treatment of invasive amebiasis.^[19]^ However, a systemic review showed that tinidazole demonstrated greater therapeutic efficacy with fewer adverse events.^[20]^ Parasite resistance to MTZ has been observed in vitro,^[21]^ which may be associated with an increased expression of iron-containing superoxide dismutase and peroxiredoxin.^[22]^ Moreover, some clinical strains of *E. histolytica* have been reported to exhibit partial resistance to MTZ, indicating the potential development of MTZ-resistant strains.^[23]^ Recent studies have investigated novel therapeutic strategies against amebiasis, promising targets including cysteine proteases, synthesis of l-cysteine, polyamine biosynthesis, and chitinase.^[24]^ In this patient, after 2 rounds of standard MTZ treatment, intestinal ulcers were still present on endoscopy, and amebic cysts could still be found in the stool until the administration of diloxanide. This indicated that luminal cyst eradication was not adequately achieved by nitroimidazoles, demanding the sequential introduction of a luminal agent. A comprehensive review suggested that a combination drug therapy achieved a reduction of 60% in clinical and parasitological failure than MTZ alone for eliminating amebae.^[20]^ However, there is insufficient information to establish the most effective combination anti-amebic drug regimen. Therefore, for symptomatic amebic colitis, the drug treatment of amebiasis is termed as a 2-drug approach: tissue amebicides like nitroimidazole or MTZ to eliminate invading trophozoites,^[25]^ and luminal amebicides like paromomycin or diloxanide to eradicate intraluminal carriage of cysts.^[26]^ For asymptomatic patients, the administration of only luminal amebicides is enough.^[27]^

## 4. Conclusion

In conclusion, any individual receiving a fresh diagnosis of IBD must undergo essential screening for amebiasis, especially in patients with recent travel history to endemic zones; long medicine history of immunosuppressants; or comorbidities like diabetes mellitus, tuberculosis, alcoholism, and pregnancy.^[28]^ Recurrent endoscopy examinations and dynamic follow-up are of great importance. For patients with IBD-like symptoms, even if endoscopic findings suggest IBD, or the results of fecal microscopy are repeatedly negative, or no trophozoites are observed in mucosal biopsies, the administration of corticosteroid should be given serious consideration, especially in cases where the effect of mesalamine is limited, but there is no significant clinical progression. When the treatment response is limited, concurrent IBD or extraintestinal amebic diseases need to be considered. For symptomatic intestinal amebiasis, after the supply of tissue amebicides like MTZ, luminal amebicides like paromomycin or diloxanide should still be used to prevent recurrence.

## Author contributions

**Investigation:** Hong Lv, Weixun Zhou, Baotong Zhou, Qingwei Jiang, Jiaming Qian.

**Writing—original draft:** Yupei Shao, Hong Lv.

**Writing—review & editing:** Yupei Shao, Hong Lv, Weixun Zhou.
